# Supplementary material for: Mapping the evolution of fertility support policies in China: A content and instrumental analysis
Source: PLoS One. 2025 Oct 9;20(10):e0332137. doi: 10.1371/journal.pone.0332137 (PMC12510515; doi:10.1371/journal.pone.0332137)
Supplement: S1 Appendix — (ZIP) [file pone.0332137.s001.zip › S1 Appendix. 226 original policy documents/207-第十三届全国人民代表大会第二次会议关于2018年国民经济和社会发展计划执行情况与2019年国民经济和社会发展计划的决议(FBM-CLI-1-330264).doc]

第十三届全国人民代表大会第二次会议关于2018年国民经济和社会发展计划执行情况与2019年国民经济和社会发展计划的决议

**发布部门：** [全国人民代表大会](https://www.pkulaw.com/law/chl?Aggs.IssueDepartment=101)

**发布日期：**2019.03.15

**实施日期：**2019.03.15

**时效性：** [现行有效](https://www.pkulaw.com/law/chl?Aggs.TimelinessDic=01)

**效力级别：** [工作文件](https://www.pkulaw.com/law/chl?Aggs.EffectivenessDic=XA0105)

**法规类别：** [人大议事](https://www.pkulaw.com/law/chl?Aggs.Category=00106) [营商环境优化](https://dobusiness.pkulaw.com/)

**专题分类：** [长江经济带发展](https://www.pkulaw.com/law/chl?Aggs.SpecialType=022) [长三角一体化发展](https://www.pkulaw.com/law/chl?Aggs.SpecialType=024)

第十三届全国人民代表大会第二次会议关于2018年国民经济和社会发展计划执行情况与2019年国民经济和社会发展计划的决议
（2019年3月15日第十三届全国人民代表大会第二次会议通过）

　　第十三届全国人民代表大会第二次会议审查了国务院提出的《关于2018年国民经济和社会发展计划执行情况与2019年国民经济和社会发展计划草案的报告》及2019年国民经济和社会发展计划草案，同意全国人民代表大会财政经济委员会的审查结果报告。会议决定，批准《关于2018年国民经济和社会发展计划执行情况与2019年国民经济和社会发展计划草案的报告》，批准2019年国民经济和社会发展计划。

　　关于2018年国民经济和社会发展计划执行情况与2019年国民经济和社会发展计划草案的报告
--2019年3月5日在第十三届全国人民代表大会第二次会议上
国家发展和改革委员会

　　各位代表：
　　受国务院委托，现将2018年国民经济和社会发展计划执行情况与2019年国民经济和社会发展计划草案提请十三届全国人大二次会议审议，并请全国政协各位委员提出意见。
　　**一、**2018年国民经济和社会发展计划执行情况
　　2018年，面对错综复杂的国际环境和艰巨繁重的国内改革发展稳定任务，在以习近平同志为核心的党中央坚强领导下，各地区各部门以习近平新时代中国特色社会主义思想为指导，深入贯彻党的十九大和十九届二中、三中全会精神，增强“四个意识”，坚定“四个自信”，做到“两个维护”，按照党中央、国务院决策部署，统筹推进“五位一体”总体布局，协调推进“四个全面”战略布局，坚持稳中求进工作总基调，贯彻新发展理念，落实高质量发展要求，以供给侧结构性改革为主线，认真执行十三届全国人大一次会议审议批准的《政府工作报告》、2018年国民经济和社会发展计划，落实全国人大财政经济委员会的审查意见、十三届全国人大常委会第七次会议对“十三五”规划《纲要》实施中期评估报告的审议意见等，大力推进市场化改革、高水平开放，大力推进现代化经济体系建设，坚决打好防范化解重大风险、精准脱贫、污染防治三大攻坚战，有效应对外部环境深刻变化，统筹稳增长、促改革、调结构、惠民生、防风险，着力做好稳就业、稳金融、稳外贸、稳外资、稳投资、稳预期工作，经济保持总体平稳、稳中有进，全年经济社会发展主要目标任务较好完成，计划执行情况总体良好。
　　（一）着力创新和完善宏观调控，经济运行保持在合理区间。更好发挥国家发展规划的战略导向作用，保持宏观经济政策的连续性和稳定性，坚决不搞“大水漫灌”式强刺激，在区间调控的基础上加强定向调控、精准调控、相机调控，主动预调微调、强化政策协同、做好预期管理，稳妥应对中美经贸摩擦，保持了经济平稳健康发展。
　　一是宏观调控目标较好完成。国内生产总值达到90.03万亿元，增长6.6%，符合预期目标。实施更加积极的就业政策，建立完善稳就业应急机制，创新创业带动就业的作用进一步增强，全年城镇新增就业1361万人，全国城镇调查失业率稳定在5%左右的较低水平。加强价格监测分析预警调控，做好市场保供稳价工作，全年居民消费价格温和上涨2.1%。国际收支基本平衡，外汇储备稳定在3万亿美元以上。
　　

　　图表：
　　关于2018年国民经济和社会发展计划执行情况与2019年国民经济和社会发展计划草案的报告 新华社发
　　

　　图表：
　　关于2018年国民经济和社会发展计划执行情况与2019年国民经济和社会发展计划草案的报告 新华社发
　　二是财政金融运行基本平稳。积极的财政政策聚力增效，减税降费力度进一步加大，财政支出结构持续优化，有力保障民生等重点领域资金需求。全国一般公共预算收入18.34万亿元，增长6.2%；全国一般公共预算支出22.09万亿元，增长8.7%；财政赤字2.38万亿元，与预算持平。稳健的货币政策保持中性，通过差别化准备金、差异化信贷等政策，引导资金更多投向民营企业和小微企业等实体经济。国家融资担保基金设立运作。年末广义货币（M2）余额增长8.1%。
　　三是投资补短板力度持续加大。充分发挥中央预算内投资对优化供给结构的引导带动作用，建立加大基础设施领域补短板力度协调机制，加强重大项目储备，统筹推进重大基础设施规划建设，铁路营业里程超过13.1万公里，其中高速铁路超过2.9万公里；高速公路总里程达到14.3万公里。促进民间投资持续健康发展，在铁路、民航、油气、电信等领域向民间资本推出一批有吸引力的项目，规范有序推广政府和社会资本合作（PPP）模式。固定资产投资（不含农户）增长5.9%，其中民间投资增长8.7%。投资结构持续优化，高技术制造业、装备制造业投资分别增长16.1%和11.1%。
　　

　　图表：
　　关于2018年国民经济和社会发展计划执行情况与2019年国民经济和社会发展计划草案的报告 新华社发
　　四是消费提质扩容积极推进。[完善促进消费体制机制进一步激发居民消费潜力的若干意见](https://www.pkulaw.com/chl/50dd2ac3d611cf7dbdfb.html)和三年实施方案出台实施，放心消费创新活动深入推进，消费环境持续优化。降低药品、汽车、日用消费品等进口关税，调整完善新能源汽车推广应用财政补贴政策。加快提升新型信息产品供给体系质量，信息消费保持快速增长。海南省建设国际旅游消费中心的实施方案、促进乡村旅游发展提质升级行动方案（2018年－2020年）印发实施，引导城乡居民扩大文化消费试点工作持续推进，上千家重点国有景区门票价格下降，全国家政培训提升行动启动，相关领域消费潜力进一步释放，全年社会消费品零售总额增长9%。消费对经济增长拉动作用增强，最终消费支出贡献率提高到76.2%。
　　

　　图表：
　　关于2018年国民经济和社会发展计划执行情况与2019年国民经济和社会发展计划草案的报告 新华社发
　　（二）供给侧结构性改革深入推进，高质量发展取得积极进展。着力进行推动高质量发展的顶层设计，制定实施推动高质量发展的意见，推动高质量发展的制度体系逐步建立。扎实推进供给侧结构性改革，以“破、立、降”为主攻方向，持续改善供给结构，发展质量效益稳步提升。
　　一是去产能工作扎实开展。结构性去产能、系统性优产能持续推进，压减粗钢产能3500万吨以上、退出煤炭落后产能2.7亿吨，均提前两年完成“十三五”目标任务，一大批“散乱污”企业出清，工业产能利用率处在较高水平。进一步做好“僵尸企业”及去产能企业债务处置工作，稳妥推进去产能职工安置，转岗再就业工作平稳有序进行。
　　二是振兴实体经济迈出新步伐。制造强国建设持续推进，工业增加值突破30万亿元。[促进首台（套）重大技术装备示范应用的意见](https://www.pkulaw.com/chl/286edf8517079f7ebdfb.html)印发实施，关键核心技术攻关加快开展。增强制造业核心竞争力三年行动计划（2018－2020年）、工业互联网发展行动计划（2018－2020年）深入实施，推动互联网、大数据、人工智能和实体经济深度融合，推进工业互联网+智能制造集成应用示范，轨道交通、高端医疗器械、工业机器人等制造业重点领域关键技术加快突破并实现产业化。新一轮技术改造升级工程组织实施，高端装备、智能制造、新材料等重点领域技术改造加快推进。质量强国战略深入实施，标准质量品牌建设持续加强。国家物流枢纽布局和建设规划印发实施。服务业创新发展大纲深入落实，服务质量提升专项行动加快推进，服务业综合改革试点深入开展。
　　

　　图表：
　　关于2018年国民经济和社会发展计划执行情况与2019年国民经济和社会发展计划草案的报告 新华社发
　　三是降成本工作持续推进。大力实施减税降费，全年为企业和个人减负约1.3万亿元。制造业等行业增值税税率从17%降至16%，交通运输、邮政、建筑、基础电信服务等行业及农产品等货物的增值税税率从11%降至10%，提高小规模纳税人年销售额标准，研发费用加计扣除比例提高到75%的政策扩大至所有企业，金融机构免征增值税贷款单户授信额度提高到1000万元。降低、停征一批行政事业性收费和政府性基金。延长阶段性降低社会保险费率和企业住房公积金缴存比例政策期限，清理规范经营服务性收费。大力推动降低用能、物流等成本，市场化交易电量比重进一步提高，全国一般工商业电价平均降低10%的目标超额完成，对挂车减半征收车辆购置税，扩大高速公路差异化收费试点。
　　（三）扎实打好三大攻坚战，重点任务取得积极进展。制定三大攻坚战行动方案，依法依规有序实施，金融治理效果明显，脱贫攻坚完成年度目标，生态环境状况明显好转。
　　一是重大风险总体可控。宏观杠杆率过快增长势头得到有效遏制，金融市场总体平稳，人民币汇率保持合理稳定，市场约束逐步增强，审慎经营理念得到强化，金融监管制度进一步完善，金融乱象整治取得阶段性成效。稳步推进外债登记制管理改革，有效防范外债风险。重点城市新建商品住宅销售价格上涨态势基本得到控制，因地制宜调整完善棚户区改造的货币化安置政策。
　　二是精准扶贫精准脱贫有力推进。进一步加大对“三区三州”等深度贫困地区和特殊贫困群体的帮扶力度，易地扶贫搬迁、以工代赈等重点任务扎实推进，产业、就业、教育、健康、生态、金融、网络、文化和旅游等扶贫工作持续深化，贫困地区自我发展能力不断增强。农村贫困人口脱贫1386万人，280万人易地扶贫搬迁建设任务顺利完成，帮扶贫困劳动力实现就业388万人。
　　三是污染防治攻坚战取得重大进展。深入贯彻习近平生态文明思想和全国生态环境保护大会精神，全面加强生态环境保护的顶层设计进一步完善，中央生态环境保护督察“回头看”有序开展，河长制湖长制全面建立，国家公园体制试点稳步推进。蓝天、碧水、净土保卫战全面推进，打赢蓝天保卫战三年行动计划等出台实施，细颗粒物（PM2.5）未达标地级及以上城市年均浓度下降10.4%，水、土壤污染防治行动深入开展，主要污染物排放总量和单位国内生产总值二氧化碳排放量进一步下降。坚定不移推进禁止洋垃圾入境，全国固体废物进口总量比上年减少46.5%。加快推进垃圾分类处理。初步划定京津冀等15个省份生态保护红线，山西等16个省份基本形成划定方案。深化生态环境保护综合行政执法改革的指导意见印发实施。重大生态保护修复工程有序推进。实施退耕还林还草1238万亩，实施草原围栏、退化草原改良等退牧还草工程3700多万亩。加强滨海湿地保护、严格管控围填海。市场化、多元化生态保护补偿机制建设积极推进，[创新和完善促进绿色发展价格机制的意见](https://www.pkulaw.com/chl/8932fd42b5f66e10bdfb.html)出台实施。北方地区冬季清洁取暖工作稳妥推进，天然气产供储销体系建设取得积极进展。重点地区煤炭消费减量替代持续推进，用能权交易试点启动实施，提前两年完成燃煤电厂超低排放和节能改造总量目标任务，清洁能源消纳长效机制建设和运行调节加强，弃电量和弃电率实现“双降”，单位国内生产总值能耗下降3.1%。全国碳排放权交易市场建设稳步推进。推动联合国气候变化大会取得积极成果，为《巴黎协定》实施细则的通过发挥重要作用。
　　（四）全面实施创新驱动发展战略，创新创业活力持续释放。强化创新第一动力作用，科技体制改革取得实质性突破，国家创新体系效能大幅提升，高技术产业和战略性新兴产业实现较快发展，“互联网+”行动深入推进，新动能培育取得积极进展，创新创业水平进一步提高，创新型国家建设迈出新步伐。
　　一是科技实力和创新能力不断提升。全国研究与试验发展经费投入强度达到2.18%，科技进步贡献率预计提高到58.5%。基础研究进一步加强，首次在半导体量子点体系中实现三量子比特逻辑门，首次发现铁基超导体中的马约拉纳束缚态，首次人工创建单条染色体真核细胞。一批重大科技成果涌现，嫦娥四号探测器成功着陆月球背面，第二艘航母出海试航，国产大型水陆两栖飞机水上首飞，北斗导航系统面向全球开启服务，5G技术系统设备达到预商用水平。重大科技、民用空间、信息等领域基础设施建设提质提速，科研管理机制和评价激励制度不断完善。全面创新改革试验169项先行先试改革举措已完成123项，第二批共23项改革举措正在复制推广。北京、上海全球影响力科技创新中心建设加速推进，粤港澳大湾区国际科技创新中心建设方案印发实施，北京怀柔、上海张江、安徽合肥3个综合性国家科学中心建设进展顺利，海南省创新驱动发展战略实施方案出台实施。国家级新区、开发区和20个国家自主创新示范区、168个国家高新区的引领作用增强，高速列车、新能源汽车等国家技术创新中心建设深入推进。国家产业创新中心工作指引出台实施，先进计算、先进存储、生物育种等3个国家产业创新中心加快建设。企业技术创新主体地位不断提高，新认定111家国家企业技术中心。
　　

　　图表：
　　关于2018年国民经济和社会发展计划执行情况与2019年国民经济和社会发展计划草案的报告 新华社发
　　二是大众创业万众创新纵深推进。[推动创新创业高质量发展打造“双创”升级版的意见](https://www.pkulaw.com/chl/8cfe8d5aea8ad43abdfb.html)出台实施，制造业“双创”平台培育三年行动计划深入推进，确定150个制造业“双创”平台试点示范项目。6500多家众创空间服务创业团队40万家，创业就业人数超过140万人。1824家星创天地培训农村创业人才4.9万人次，孵化企业2.3万家。加强“双创”示范基地建设和小微企业创业创新示范基地建设，建立完善国家创新创业政策信息服务网，成功举办2018年全国“双创”活动周和“创响中国”系列活动。国家新兴产业创业投资引导基金有效运转，新投资企业超过1200家。全年新登记注册企业增长10.3%，平均每天新设1.84万户。
　　

　　图表：
　　关于2018年国民经济和社会发展计划执行情况与2019年国民经济和社会发展计划草案的报告 新华社发
　　三是新兴产业蓬勃发展。全面落实“十三五”国家战略性新兴产业发展规划，推动战略性产业集群发展，“互联网+”、新一代信息基础设施、人工智能创新发展、生物产业倍增、民用空间基础设施等重大工程加速推进。规模以上高技术制造业增加值增长11.7%，快于全部规模以上工业增速。[电子商务法](https://www.pkulaw.com/chl/3f020f79c1e5316ebdfb.html)正式公布，引导和规范数字经济、共享经济等新业态健康发展的政策文件陆续出台，对新业态新模式的包容审慎监管进一步完善，智能零售、产能共享等新热点持续涌现，全年实物商品网上零售额增长25.4%，占社会消费品零售总额比例达18.4%，比上年提高3.4个百分点。京津冀、贵州等8个国家大数据综合试验区加快建设。
　　四是军民融合发展战略扎实推进。国家军民融合创新示范区启动建设。科技军民融合重点专项与军民科技协同创新平台启动，军民融合重大示范项目建设进展顺利。国家军民融合信息化平台建设有力推进。国家国防科技工业军民融合产业投资基金、国家级军民融合发展产业投资基金启动运行。支持符合条件的军民融合企业发行企业债券，拓宽军民融合投融资渠道。第三届中国军民两用技术创新应用大赛和第四届军民融合高技术装备成果展成功举办。
　　（五）深化改革扩大开放，经济社会发展动力进一步激发。按照深化党和国家机构改革方案，国务院机构改革有序推进。深入开展纪念改革开放40周年系列庆祝活动，推动一系列重大改革开放举措落地。
　　一是营商环境持续优化。“放管服”改革深入推进。全面实施全国统一的[市场准入负面清单](https://www.pkulaw.com/chl/d8135b05437494cebdfb.html)制度，[市场准入负面清单](https://www.pkulaw.com/chl/d8135b05437494cebdfb.html)（2018年版）发布，清单以外的行业、领域、业务等各类市场主体皆可依法平等进入。取消汽车投资项目核准等一批行政许可事项，大幅压缩企业开办时间，副省级以上城市和省会城市企业开办时间压缩至8.5个工作日以内。“证照分离”改革在全国推开，全面实施全国统一“二十四证合一”改革。工业产品生产许可证制度改革加快推进，产品种类由38类降至24类。在北京等15个城市和浙江省开展工程建设项目审批制度改革试点。世界银行公布的我国营商环境全球排名由2017年第78位大幅跃升至第46位。在22个城市开展中国营商环境试评价，中国营商环境评价体系初步构建。深入推进“双随机、一公开”监管，基本实现市场监管领域日常监管全覆盖。社会信用体系建设持续推进，电信诈骗、偷逃骗税等19个重点领域失信问题专项治理深入开展，守信联合激励和失信联合惩戒机制初显成效。政务服务“一网一门一次”改革启动实施，建立国务院部门数据共享责任清单，基本建成全国一体化的数据共享交换平台体系，数据共享交换量达360亿条次。深入推进审批服务便民化，“减证便民”行动和“群众办事百项堵点疏解行动”扎实开展。
　　二是国资国企改革加快推进。国有企业混合所有制改革积极稳步开展，深化混合所有制改革试点有关政策出台实施，三批50家混合所有制改革试点梯次推进并取得重要阶段性成效。[改革国有企业工资决定机制的意见](https://www.pkulaw.com/chl/0004395df5d25cf6bdfb.html)、[推进国有资本投资、运营公司改革试点的实施意见](https://www.pkulaw.com/chl/b981aac4a885ffa9bdfb.html)出台，深化国企改革“1+N”政策体系搭建完成。加强国有企业资产负债约束的指导意见印发实施，国有企业资产负债约束机制初步建立。中国特色现代国有企业制度进一步完善，有效制衡的法人治理结构和灵活高效的市场化经营机制加快建立。
　　

　　图表：
　　关于2018年国民经济和社会发展计划执行情况与2019年国民经济和社会发展计划草案的报告 新华社发
　　三是支持民营经济发展力度加大。加强产权和知识产权保护，建立健全产权保护协调机制，全面清理涉及产权保护的规章规范性文件，深入开展政府机构失信问题专项治理行动。“互联网+”知识产权保护工作方案出台实施。大力弘扬企业家精神，[私营企业暂行条例](https://www.pkulaw.com/chl/2ea1407c16fd5803bdfb.html)废止。着力疏通货币政策传导机制，鼓励金融机构扩大对实体经济特别是制造业企业、民营企业、小微企业的信贷投放。设立民营企业债券融资支持工具，以市场化方式帮助企业缓解融资难问题。
　　四是财税金融改革稳步推进。中央与地方财政事权和支出责任划分改革分领域推进，预算绩效管理制度不断完善。增值税改革进一步深化。修改[个人所得税法](https://www.pkulaw.com/chl/d74fa423af05cffdbdfb.html)及其实施条例，提高基本减除费用标准，设立6项专项附加扣除，税率结构进一步调整优化。环境保护税顺利开征。完善系统重要性金融机构监管、加强非金融企业投资金融机构监管、完善国有金融资本管理等政策文件出台实施。有序推动民营银行发展，17家民营银行获批开业。
　　

　　图表：
　　关于2018年国民经济和社会发展计划执行情况与2019年国民经济和社会发展计划草案的报告 新华社发
　　五是共建“一带一路”取得新进展。推进“一带一路”建设工作5周年座谈会召开，为推动“一带一路”建设走深走实明确了方向。围绕重点方向重点国别，巩固共建意愿、推动规划对接，已累计同150多个国家和国际组织签署171份政府间合作文件。国际产能合作稳步推进，境外经贸合作区产业集聚效应增强，与法国、日本、新加坡等10多个国家签署第三方市场合作文件，中老、中泰铁路和中阿（联酋）“一带一路”产能合作园区等重点项目取得实质性进展，雅万高铁全面开工，瓜达尔港等重点港口项目进展顺利，中欧班列提质增效工作取得重要进展，累计开行超过1.3万列，回程率提高近20个百分点。“一带一路”沿线国家航空互联互通水平显著提高，新开航线106条。“一带一路”国际科学组织联盟成立，推动与东盟、南亚、阿拉伯国家、中亚、中东欧5个区域共建技术转移平台，推动与菲律宾、印尼等8个国家共建科技园区。中非合作论坛北京峰会取得圆满成功和丰硕成果。数字丝绸之路建设稳步推进，已与16个国家签署合作谅解备忘录，“丝路电商”全球布局步伐加快，与17个国家签署合作协议。
　　

　　图表：
　　关于2018年国民经济和社会发展计划执行情况与2019年国民经济和社会发展计划草案的报告 新华社发
　　六是贸易强国建设扎实推进。首届中国国际进口博览会成功举办，向全世界宣示了我国主动开放市场、推动经济全球化的决心。区域全面经济伙伴关系协定谈判加速推进，与新加坡签署自贸协定升级版，与毛里求斯完成自贸协定谈判，累计已与25个国家和地区达成17个自贸协定，多双边经贸合作进一步深化。出台53项措施支持自贸试验区深化改革创新，在全国范围内复制推广30项自贸试验区改革试点经验。海南全岛自贸试验区启动建设，探索建设中国特色自由贸易港。新设一批跨境电子商务综合试验区，完善跨境电商零售进口监管和税收政策，扩大市场采购贸易试点范围，跨境电子商务、市场采购贸易等新业态新模式蓬勃发展。关税总水平由9.8%下调至7.5%，积极扩大进口促进对外贸易平衡发展。多元化市场不断拓展，全年货物贸易进出口总额达30.51万亿元，增长9.7%。
　　

　　图表：
　　关于2018年国民经济和社会发展计划执行情况与2019年国民经济和社会发展计划草案的报告 新华社发
　　七是利用外资环境不断优化。全面放宽市场准入，2018年版全国和自贸试验区两个外资准入负面清单发布，特别管理措施分别压减至48条、45条，金融领域对外开放稳步推进。在全国推行外资企业设立商务备案与工商登记“一口办理”，积极推进在产业政策、科技政策、政府采购、资质许可、标准制定等方面平等对待内外资企业，开发区改革和创新发展不断深化，外商投资环境持续优化。全年外商直接投资达1350亿美元，增长3%。
　　八是对外投资平稳有序发展。[企业境外投资管理办法](https://www.pkulaw.com/chl/411abaaa11209afcbdfb.html)、对外投资备案（核准）报告暂行办法正式施行，全口径、全过程管理不断完善，对外投资行业结构进一步优化，主要流向租赁和商务服务业、制造业等。发布企业境外经营合规管理指引，企业合规意识有所增强。引导对外投融资基金健康发展，企业“走出去”融资渠道进一步拓宽。全年非金融类境外直接投资达到1205亿美元。
　　（六）大力实施乡村振兴战略，农业农村发展新动能加快培育。乡村振兴战略规划（2018－2022年）出台实施，28个省级乡村振兴战略规划已印发，其他省（区、市）已基本完成规划编制工作，多规合一的村庄规划编制部署推进，乡村振兴的制度框架和政策体系逐步建立，乡村振兴阶段性重点工作全面展开。
　　一是农业基础进一步夯实。粮食总产量保持在1.3万亿斤以上。新增高效节水灌溉面积2158万亩，新增高标准农田8000多万亩，完成9亿多亩粮食生产功能区和重要农产品生产保护区划定任务。深入实施现代化种业提升工程，高标准建设国家南繁科研育种基地，加快推动粮油、蔬菜、水果等农作物种子种苗基地建设。172项重大水利工程累计开工133项，在建投资规模超过1万亿元。全年完成营造林2.34亿亩。开展“农业质量年”行动，加快推进质量兴农、绿色兴农、品牌强农，主要农产品质量安全监测合格率保持在97%以上，化肥、农药使用量均下降。
　　二是农村一二三产业融合发展取得积极进展。认定农村产业融合发展示范园100家，启动创建现代农业产业园62个、农业产业强镇254个。深入实施主要农作物生产全程机械化推进行动。大力发展智慧农业，农业生产、经营、管理、服务数字化水平进一步提升。农村新产业新业态加快培育。
　　三是农业农村改革稳步推进。完善稻谷、小麦最低收购价政策制度。健全玉米和大豆市场化收购加补贴机制。农村土地征收、集体经营性建设用地入市、宅基地制度改革试点工作有序推进。宅基地和农房确权登记发证加快实施，土地承包权确权登记颁证工作基本完成，承包地已确权面积14.8亿亩，超过二轮家庭承包地（账面）面积。建立健全进城落户农民土地承包权、宅基地使用权、集体收益分配权维护和自愿有偿退出机制，在3个省、50个地市、150个县启动第三批农村集体产权制度改革试点。全国已有超过15万个农村集体组织完成改革，共确认集体成员2亿多人。集体林权制度改革进一步深化。
　　四是美丽宜居乡村建设步伐加快。农村人居环境整治三年行动全面启动。农村突出环境问题综合治理试点示范和农村生活污水治理示范有序开展。农村生活垃圾治理覆盖面进一步扩大，厕所革命积极推进。农村饮水安全巩固提升工程加快实施，农村自来水普及率进一步提升。农村公路建设加快推进。农村机井通电、小城镇中心村农网改造升级、贫困村通动力电等顺利完成。开展电信普遍服务试点、“宽带乡村”、“百兆乡村”示范工程，新一代信息基础设施建设工程加快推进。农村基层综合性文化服务中心建设有序推进。
　　（七）统筹推动区域协调发展，空间发展格局更趋优化。发挥各地区比较优势，努力缩小区域发展差距，大力推进新型城镇化建设，区域发展的协同性、联动性、整体性进一步增强。
　　一是进一步促进区域协调发展。[建立更加有效的区域协调发展新机制的意见](https://www.pkulaw.com/chl/e89120dfd41e7583bdfb.html)出台实施。推动西部地区进一步开发开放，新开工交通、能源等重点工程28项，总投资4825亿元。东北地区营商环境建设、民营经济发展、与东部对口合作等取得新突破，老工业城市和资源型地区转型稳步推进，城区老工业区、独立工矿区改造搬迁和采煤沉陷区综合治理持续实施。先进制造业在中部地区合理布局提速，汉江、淮河生态经济带发展规划以及湘南湘西承接产业转移示范区总体方案出台实施。东部地区新动能加快培育壮大，组织开展山东新旧动能转换综合试验区建设。革命老区、民族地区、边疆地区、贫困地区加快发展，交通、水利、能源、通信、物流等重大基础设施建设扎实推进，对口支援深入开展，支持新疆、西藏和四省藏区经济社会发展政策措施进一步完善。海洋经济发展示范区启动建设，海洋强国建设加快推进。国家级新区、临空经济示范区、综合配套改革试验区等功能平台的示范引领作用不断增强。沿边重点开发开放试验区建设稳步推进，形成了一批可复制可推广经验。
　　二是重大区域发展战略有力推进。以疏解北京非首都功能为“牛鼻子”，推动京津冀协同发展取得明显进展，[河北雄安新区规划纲要](https://www.pkulaw.com/lar/0429026816a72f964897a6f66c7c926ebdfb.html)、[支持河北雄安新区全面深化改革和扩大开放的指导意见](https://www.pkulaw.com/chl/c7df71496dd4a759bdfb.html)、河北雄安新区总体规划（2018－2035年）出台实施，北京城市副中心规划建设提速，京雄城际铁路开工建设，北京大兴国际机场建设和运营筹备工作全面推进。长江经济带共抓大保护格局进一步巩固，国土空间规划编制工作启动，“三水共治”、河湖清“四乱”专项行动扎实推进，1361座非法码头专项整治全面完成，长江干流断面水质优良比例明显提高。综合立体交通走廊建设加快推进，沿江高铁规划建设、省际待贯通路段建设提速、长江深水航道建设及干线港口铁水联运取得积极进展。粤港澳大湾区发展规划纲要发布实施，重点项目建设全面启动，港珠澳大桥正式开通运营，一系列便利港澳居民在内地发展的政策举措落地见效。[支持海南全面深化改革开放的指导意见](https://www.pkulaw.com/chl/473d5ba43988913fbdfb.html)出台实施。长江三角洲区域一体化发展规划纲要启动编制。
　　

　　图表：
　　关于2018年国民经济和社会发展计划执行情况与2019年国民经济和社会发展计划草案的报告 新华社发
　　三是新型城镇化质量有效提升。农业转移人口进城落户更加便捷，大中城市落户政策持续放宽，居住证制度全覆盖目标基本实现，全国常住人口城镇化率达59.58%，户籍人口城镇化率达43.37%，比上年末分别提高1.06个百分点和1.02个百分点。一批跨省（区、市）城市群规划出台实施，各地城市群建设全面推进，现代化都市圈加快培育发展。
　　

　　图表：
　　关于2018年国民经济和社会发展计划执行情况与2019年国民经济和社会发展计划草案的报告 新华社发
　　（八）着力保障和改善民生，发展成果更多更公平惠及人民群众。坚持以人民为中心的发展思想，紧紧围绕人民群众普遍关心的突出问题，推动一大批惠民举措落细落实。
　　一是稳定就业有序推进。[做好当前和今后一个时期促进就业工作的若干意见](https://www.pkulaw.com/chl/54625ef77009d3c1bdfb.html)、[推行终身职业技能培训制度的意见](https://www.pkulaw.com/chl/90ee278ef064570abdfb.html)出台实施。公共就业创业服务持续加强，援企稳岗力度加大，支持农民工等人员返乡创业试点工作持续推进。高校毕业生就业创业促进计划等深入实施。制定出台推进全方位公共就业服务指导意见，“互联网+公共就业服务”全面推进。[人力资源市场暂行条例](https://www.pkulaw.com/chl/381230d756f4e881bdfb.html)发布实施，人力资源市场体系逐步健全。
　　二是社会保障制度日益完善。企业职工基本养老保险基金中央调剂制度正式实施，城乡居民基本养老保险待遇确定和基础养老金正常调整机制建立。统一社保费征收管理机构工作稳步推进，社保费征收管理制度进一步规范。整合城乡居民基本医疗保险制度持续推进。养老保险覆盖人数已超过9.42亿人，基本医疗保险覆盖人数已超过13.44亿人。国家跨省异地就医结算平台进一步完善，跨省定点医疗机构实现县级行政区全覆盖，外出农民工、外来就业创业人员全部纳入覆盖范围，异地就医直接结算累计达152.6万人次。生育保险与职工医保合并实施试点稳步推进。失业保险“保生活、防失业、促就业”功能不断完善。农民工参加工伤保险政策进一步健全。低保制度不断完善，城乡低保实现全覆盖且保障水平持续提高。退役士兵就业安置进一步加强，带病回乡退伍军人、参战参试退役人员等优抚对象生活补助标准提高。社会福利服务体系建设加强，社会救助综合改革试点有序开展。城镇保障性安居工程建设扎实推进，城镇棚户区住房改造开工达626万套，公租房新增分配100万套，大中城市住房租赁市场加快培育。农村危房改造190万户。
　　三是基本公共服务标准体系加快构建。建立健全基本公共服务标准体系的指导意见出台实施，基本公共服务省级清单实现全覆盖。教育现代化推进、全民健康保障、文化旅游提升、公共体育普及、社会服务兜底五大公共服务工程大力推进。九年义务教育巩固率达94.2%，高中阶段教育毛入学率达88.8%，财政性教育经费支出占国内生产总值比例继续超过4%。“互联网+医疗健康”快速发展，公立医院综合改革继续深化，国家基本药物制度进一步完善，药品集中采购和使用试点稳妥推进，境外上市新药审评审批加快，进口抗癌药全部实现零关税，17种抗癌药大幅降价并纳入医保报销目录，疫苗药品安全问题加快解决。每千人口医疗卫生机构床位数预计达6.05张。加强文物保护利用改革的若干意见印发实施，各类遗产资源保护状态得到有效改善。大运河文化保护传承利用规划纲要编制完成。公共文化设施免费开放工作持续开展，百万公里健身步道工程实施方案出台，新增体育场地8.2万个。社会兜底保障加强，困难残疾人生活补贴制度惠及940万人，1164万重度残疾人享受护理补贴。孤儿保障制度惠及39.8万孤儿，为全国残疾孤儿实施医疗康复。持续开展孕期优生优育服务，妇女关爱服务体系不断健全。公共法律服务体系继续完善。全年出生人口1523万人，年末总人口达13.95亿人，人口自然增长率为3.81‰。
　　

　　图表：
　　关于2018年国民经济和社会发展计划执行情况与2019年国民经济和社会发展计划草案的报告 新华社发
　　总的来看，2018年经济社会发展主要目标任务较好完成，三大攻坚战开局良好，供给侧结构性改革深入推进，改革开放力度加大，人民生活持续改善，保持了经济平稳健康发展和社会大局稳定，朝着实现全面建成小康社会的目标迈出了新的步伐。在极为错综复杂的国内外形势下，我国经济社会发展取得这样的成绩来之不易，需要倍加珍惜。这是以习近平同志为核心的党中央举旗定向、谋篇布局、坚强领导的结果，是习近平新时代中国特色社会主义思想全面引领、科学指导、成功实践的结果，是全党全国各族人民团结一心、砥砺前行、真抓实干的结果。
　　从2018年经济社会发展情况看，经济增长、就业、价格总水平、国际收支平衡等主要指标保持在合理区间，创新驱动、资源节约、生态保护、社会保障等反映高质量发展的指标进一步改善，19个约束性指标中，18个指标完成全年目标，万元国内生产总值用水量下降指标完成情况与全年目标存在差距。该指标2018年计划目标为下降5.2%，初步统计数为下降5.1%，低于计划目标0.1个百分点。主要原因是，2018年全国降水量下降使得地表水资源总量减少约800亿立方米，部分地区受干旱影响，农业从河流抽取的灌溉用水量增加；南水北调工程向华北地区进行生态补水，全国生态用水量较2017年有较大增加，综合因素使得万元国内生产总值用水量降幅低于全年目标0.1个百分点。
　　46个预期性指标中，44个指标运行情况符合或好于年度预期目标，但社会融资规模存量增长率和城镇居民人均可支配收入指标实际运行值与预期值存在一定差距。关于社会融资规模存量增长率，该指标2018年预期目标为“与2017年实际增速基本持平”，2017年末社会融资规模存量增长13.4%，2018年末实际增长9.8%，与预期目标存在一定差距。为了给供给侧结构性改革营造适宜的货币金融环境，在对实体经济提供稳定融资支持的同时，主动采取了规范地方政府隐性债务以及合理控制投向房地产、环评不达标企业、产能过剩等领域的资金和抑制资金脱实向虚、减少资金空转等措施，社会融资规模存量增长率虽低于预期目标，但与名义GDP增长基本匹配。关于城镇居民人均可支配收入增速，该指标2018年预期目标为“与经济增长基本同步”，2018年GDP增长6.6%，城镇居民人均可支配收入实际增长5.6%，与预期目标存在一定差距。主要是受经济下行压力加大、实体经济特别是小微企业困难增多、部分行业企业利润有所下滑等影响，工资性收入等增长放缓，使得全国城镇居民人均可支配收入增速低于预期目标。
　　同时，我们也清醒认识到，经济运行总体平稳、稳中有进，但稳中有变、变中有忧。从国际形势看，世界大变局加速深刻演变，全球动荡源和风险点增多，我国外部环境复杂严峻。世界经济虽有望延续复苏态势，但保护主义、单边主义加剧，主要发达经济体货币政策调整的外溢效应持续显现，部分新兴经济体面临的风险增多，国际大宗商品价格波动加剧，地缘政治风险累积发酵，全球经济贸易增速趋缓。从国内形势看，一是经济面临下行压力。内需增长放缓，基础设施、制造业、房地产开发等领域投资保持平稳增长的难度加大。受居民增收困难较多、汽车等传统消费放缓等因素影响，消费稳定增长面临挑战。二是农业农村基础仍然薄弱。农业科技水平仍待提升，农村基本公共服务供给和基础设施建设仍然滞后，农民增收渠道有待拓宽。三是实体经济面临困难增多。民营企业特别是小微企业融资难融资贵问题尚未有效解决，存在对民营企业惜贷压贷甚至直接抽贷断贷的现象，能源、原材料、人工、用地等成本较高，企业盈利空间受到挤压。营商环境有待进一步优化，公平竞争的市场机制尚不完善，产权和知识产权保护仍不充分，亲清新型政商关系仍不健全。四是高质量发展的短板制约仍待突破。重大原创性科技成果不多，一些关键核心技术受制于人，科技成果转化效率不高。产业结构还需进一步优化，发展新动能仍然不足。部分地区能源资源约束趋紧，生态环境问题依然突出。五是区域分化态势仍在持续。中西部一些地区结构调整难度较大，一些地区要素吸引力较弱，人才等优质要素资源外流比较严重，发展困难可能会增加。六是重点领域风险压力较大。外部环境不确定性可能通过贸易、跨境资本流动、大宗商品市场等渠道，加大我国输入性风险，国内股市、债市、汇市、互联网金融和房地产的风险隐患不容忽视。七是社会民生领域还存在不少问题挑战。部分省份就业结构性矛盾显现，居民持续增收制约因素增多，教育、医疗、养老等公共服务供给不足，人口老龄化程度不断加深。同时，我们在工作中还存在一些不足，有的政策制定的前瞻性、针对性、有效性还不够，没有充分考虑企业的适应程度问题；有的政策部门间统筹协调有待加强，有的改革举措和政策落实效果不彰，有的专项规划和项目建设需要及时启动；有的地方在政策执行过程中存在简单化、“一刀切”的问题。
　　对上述问题，我们将高度重视，既要增强忧患意识、坚持底线思维，抓住主要矛盾，有针对性地加以解决，有效防范各类风险连锁联动、叠加传导；又要紧扣社会主要矛盾的变化，全面用好我国发展的重要战略机遇期，保持战略定力、坚定必胜信心，化挑战为机遇、变压力为动力，加快推动经济高质量发展。

　　**二、**2019年经济社会发展总体要求、主要目标和政策取向
　　2019年是新中国成立70周年，是全面建成小康社会、实现第一个百年奋斗目标的关键之年，做好经济社会发展工作至关重要。
　　（一）总体要求。
　　2019年，要在以习近平同志为核心的党中央坚强领导下，以习近平新时代中国特色社会主义思想为指导，全面贯彻党的十九大和十九届二中、三中全会精神，统筹推进“五位一体”总体布局，协调推进“四个全面”战略布局，坚持稳中求进工作总基调，坚持新发展理念，坚持推动高质量发展，坚持以供给侧结构性改革为主线，坚持深化市场化改革、扩大高水平开放，加快建设现代化经济体系，继续打好三大攻坚战，着力激发微观主体活力，创新和完善宏观调控，统筹推进稳增长、促改革、调结构、惠民生、防风险、保稳定工作，保持经济运行在合理区间，进一步稳就业、稳金融、稳外贸、稳外资、稳投资、稳预期，提振市场信心，增强人民群众获得感、幸福感、安全感，保持经济持续健康发展和社会大局稳定，为全面建成小康社会收官打下决定性基础，以优异成绩庆祝中华人民共和国成立70周年。
　　在具体工作中，必须牢牢把握中央经济工作会议精神，深刻把握重要战略机遇新内涵，切实做到“五个坚定不移”。一是坚定不移以供给侧结构性改革为主线。更多采取改革的办法，更多运用市场化、法治化手段，在“巩固、增强、提升、畅通”八个字上下功夫，着力提高供给体系质量。二是坚定不移推动高质量发展。有效发挥高质量发展指标体系的导向作用，加快建设现代化经济体系，加大实施创新驱动发展战略力度，深入推进军民融合发展，扎扎实实抓好政策落实，久久为功增强发展韧性。三是坚定不移促进形成强大国内市场。紧扣社会主要矛盾变化，通过扎实推进高质量发展、深化供给侧结构性改革，不断释放和满足国内市场需求，促进提升中长期发展潜力，推动经济持续健康发展。四是坚定不移打好三大攻坚战。着眼全面建成小康社会全局目标，坚持底线思维，抓住主要矛盾，精准施策，攻坚克难，确保如期完成任务，切实增强群众获得感。五是坚定不移深化市场化改革扩大高水平开放。制定出台实施一批既利当前、更利长远的改革开放举措，大力增强市场主体活力，充分调动社会各方面积极性、主动性、创造性。
　　同时，着力把握好三个关系。一是统筹好国内与国际的关系，既重视应对外部变化，更注重立足国内发展，敢于应对挑战，善于化危为机，着力办好自己的事，稳妥有序解决好国内长期积累的体制性结构性矛盾；二是平衡好稳增长与防风险的关系，在稳定经济增长、推动高质量发展中应对挑战、化解风险，继续保持宏观政策的连续性稳定性，把握好防风险的节奏和力度，避免形成不利于稳预期、稳增长、调结构的叠加效应，确保经济运行在合理区间；三是处理好政府与市场的关系，更多运用改革创新的办法发挥市场在资源配置中的决定性作用，更好发挥政府作用，增强宏观政策的稳定性、有效性，进一步激发微观主体活力、增强内生动力。
　　（二）主要预期目标。
　　按照上述总体要求和工作思路，综合考虑需要和可能，提出2019年经济社会发展主要预期目标：
　　--经济增长保持在合理区间。国内生产总值增长预期目标为6%－6.5%。主要考虑：一是充分估计经济运行中的不稳定不确定因素，按照实事求是的原则，适当调整经济增长预期目标。同时，考虑到经济增速的季节波动，采用区间式预期目标增加弹性。二是保持合理的经济增长速度，目的是满足新增就业的需要，并为推动高质量发展、深化供给侧结构性改革、打好三大攻坚战提供必要的宏观环境，有利于稳定市场预期。三是这一预期目标与当前我国经济增长潜力相符，与今年一、二、三次产业增长预期目标相匹配，三次产业稳定增长可以为经济保持中高速增长提供有力支撑。
　　--经济发展质量和效益不断提升。着力巩固“三去一降一补”成果，宏观杠杆率基本稳定，财政金融风险有效防控；全员劳动生产率增长6.3%，科技进步贡献率达到59.2%，研究与试验发展经费投入强度达到2.2%；全国常住人口城镇化率达到60.6%，户籍人口城镇化率达到44.4%；生态环境进一步改善，细颗粒物（PM2.5）未达标地级及以上城市浓度下降2%，单位国内生产总值能耗降低3%左右，单位国内生产总值二氧化碳排放量下降3.6%左右，主要污染物排放量继续下降；城乡居民收入增长与经济增长基本同步，社会保障覆盖面进一步扩大，教育、卫生、文化、养老等基本公共服务均等化水平进一步提高。更加重视这些反映质量、效益和结构的指标，主要考虑是坚持新发展理念，坚持以人民为中心的发展思想，按照质量第一、效益优先的原则，更好体现高质量发展在供给、需求、投入产出、分配、宏观经济循环等方面的特征，不断提高人民群众的获得感和幸福感。
　　--就业形势总体稳定。城镇新增就业预期目标为1100万人以上；全国城镇调查失业率和城镇登记失业率预期目标分别为5.5%左右和4.5%以内。关于城镇新增就业总量：既包含了2019年新成长劳动力在城镇的就业需求，也为去产能职工安置、失业人员再就业、农村劳动力转移就业留有一定空间。从经济基本面和经济增长对就业的吸纳能力看，6%－6.5%的经济增速能够实现新增就业岗位目标。关于失业率：将全国城镇调查失业率控制在5.5%左右，主要是考虑到进出口国际经济环境不确定影响和高校毕业生增加等因素，较好体现稳就业的决心以及实施就业优先、强化就业保障的政策导向。从全国城镇登记失业率看，近年来一直保持在4%左右，按4.5%以内考虑，主要是考虑到数据的季节性波动，适当留有余地。同时，从全年来看，争取更好结果，把失业率控制在相对较低水平。
　　--价格总水平基本稳定。居民消费价格涨幅预期目标为3%左右。主要考虑：从翘尾因素看，2019年居民消费价格中的翘尾因素约为0.7个百分点。从新涨价因素看，受非洲猪瘟疫情等影响，一些食品价格可能上涨；服务价格可能保持涨势，进口大宗商品价格上行的可能性依然存在。
　　--国际收支保持基本平衡。进出口稳中提质。主要考虑：从必要性看，在风险挑战增多情况下，保持国际收支基本平衡和合理规模外汇储备，有利于稳定经济金融和市场预期。从可行性看，2019年全球经济贸易增长态势仍有望延续，我国将举办第二届“一带一路”国际合作高峰论坛和第二届中国国际进口博览会，都有利于进出口稳定增长。同时，考虑到单边主义、贸易保护主义对我国进出口的影响，特别是中美经贸摩擦的不确定性因素，要在稳定外贸基本盘的前提下，促进贸易结构优化、质量提升。
　　（三）主要政策取向。
　　宏观政策要强化逆周期调节，继续实施积极的财政政策和稳健的货币政策，适时预调微调。结构性政策要强化体制机制建设，坚持向改革要动力，深化国资国企、财税金融、土地、市场准入、社会管理等领域改革。社会政策要强化兜底保障功能，实施就业优先政策，确保群众基本生活底线，寓管理于服务之中。
　　--积极的财政政策要加力提效。2019年财政赤字率拟按2.8%安排，比上年预算高0.2个百分点；财政赤字2.76万亿元，比上年预算增加3800亿元；同时，较大幅度增加地方政府专项债券规模。实施更大规模的减税降费，深化增值税改革、实质性降低增值税税率，全面实施修改后的[个人所得税法](https://www.pkulaw.com/chl/d74fa423af05cffdbdfb.html)及其实施条例，明显降低企业社保缴费负担。调整优化财政支出结构，一般性支出压减5%以上、“三公”经费再压减3%左右，切实保障重点支出，发挥好财政资金精准补短板和民生兜底作用，增加对脱贫攻坚、“三农”、结构调整、科技创新、生态环保、民生等领域投入。更好发挥转移支付作用，继续较大幅度增加中央对地方转移支付，改革完善县级基本财力保障机制，增强困难地区和基层政府保基本民生能力。
　　--稳健的货币政策要松紧适度。广义货币（M2）和社会融资规模增速与国内生产总值名义增速相匹配，与2018年实际增速基本持平，以更好满足经济运行保持在合理区间的需要。灵活运用多种货币政策工具组合，保持流动性合理充裕和市场利率水平合理稳定。改善货币政策传导机制，加强货币政策与金融监管等协调配合，深化利率市场化改革，提高直接融资比重，有效缓解实体经济特别是制造业企业和小微企业、民营企业融资难融资贵问题。进一步完善汇率形成机制，增强人民币汇率弹性，加强跨境资本流动监管，保持人民币汇率在合理均衡水平上的基本稳定。着力保持股市、债市、汇市稳定并及时应对异常波动，防范化解金融风险隐患。
　　--就业优先政策要全面发力。把稳定和促进就业作为经济社会发展的优先目标，完善就业促进政策体系，更好发挥大众创业、万众创新对扩大就业的支撑作用。促进就业容量大的服务业和部分劳动密集型产业发展，支持民营企业、中小微企业更好发挥就业主渠道作用，促进高校毕业生、退役军人、下岗职工、农民工等群体就业，大规模开展职业技能培训，加大对灵活就业、新就业形态的支持，加强全方位公共就业服务，努力在推动经济高质量发展中实现更高质量和更充分就业。
　　--强化政策统筹协调。进一步做好顶层设计，增强政策的连续性、稳定性和协同性，把握好政策出台的时机和力度，发挥国家发展规划的战略导向作用，更加注重年度国民经济和社会发展计划与“十三五”规划《纲要》实施的衔接，健全财政、货币、产业、区域等经济政策协调机制，探索建立宏观经济政策评估机制，防止出现政策不协调不配套，避免政策效应出现负向叠加、反向对冲等情况。产业政策要坚持普惠化、功能性，加强对优化供给结构、提高供给质量的支持。强化竞争政策的基础性地位，着眼于使市场在资源配置中起决定性作用和更好发挥政府作用，加强公平竞争审查和反垄断、反不正当竞争执法，规范政府行为，打破行政垄断和地方保护，优化和完善技术、环保、质量、安全等标准，降低市场准入门槛，完善产权制度和市场退出制度，营造公平竞争的制度环境，进一步构建和完善全国统一大市场，鼓励中小企业加快成长。区域政策要加快落实区域协调发展战略，因地制宜、分类指导，发挥比较优势，引导要素有序流动，健全区域合作机制和市场一体化发展机制。加快构建城乡融合发展体制机制和政策体系。完善农业转移人口市民化政策体系，推动农民工及随迁家属平等享受城镇基本公共服务，在人口转移和城乡建设中激发内需潜力。

　　**三、**2019年国民经济和社会发展计划的主要任务
　　2019年，要以习近平新时代中国特色社会主义思想为指导，全面贯彻党的十九大和十九届二中、三中全会精神，按照党中央、国务院决策部署，深入落实中央经济工作会议精神和《政府工作报告》部署要求，着力做好八方面工作。
　　（一）着力深化供给侧结构性改革。按照“巩固、增强、提升、畅通”八字方针，巩固“三去一降一补”成果，增强微观主体活力，提升产业链水平，畅通国民经济循环，有效缓解企业困难，厚植实体经济发展根基。
　　一是推动制造业高质量发展。制定实施大力推动制造业高质量发展的部署方案，培育发展先进制造业集群。继续推进结构性去产能，健全各方面责任共担和损失分担机制，稳步推进企业优胜劣汰，加快处置“僵尸企业”，制定退出实施办法。将降低企业杠杆率与企业兼并重组、产业整合有机结合，稳妥做好去产能职工分流安置工作。
　　二是推进先进制造业与现代服务业深度融合。出台实施深化新一代信息技术与制造业融合发展的指导意见，推动工业互联网创新发展与智能制造、电子商务有机结合、互促共进，积极推动传统制造业加速向数字化、网络化、智能化发展，推进制造业物流业融合发展。制定出台服务业高质量发展行动纲要，深入开展服务业综合改革试点。
　　三是更大力度降成本。坚持普惠性减税和结构性减税相结合，对小微企业、科技型初创企业实施普惠性税收减免。启动环境保护、节能节水项目和资源综合利用企业所得税优惠目录修订工作。清理规范经营服务性收费。发挥好国家融资担保基金作用，落实好小微企业融资担保降成本奖补机制。推动公共信用信息、金融信用信息共享，创新基于信用的小微企业、“三农”融资模式。大力推广集装化运输，持续推进多式联运示范工程，着力打通公铁水联运衔接的“最后一公里”，切实降低物流成本。
　　四是加力支持民营企业发展。坚持“两个毫不动摇”，鼓励、支持、引导非公有制经济继续发展壮大，大力营造公平竞争的市场环境和法治化制度环境。持续加强产权保护，强化政务诚信建设，开展产权保护领域政务失信专项治理行动，推动各地解决一批企业和群众反映强烈的产权纠纷问题。建立健全企业家参与涉企政策制定机制，着力构建亲清新型政商关系，切实将激发和保护企业家精神各项政策落到实处。出台支持民营企业改革发展的意见。依法保护民营企业权益，有力保护企业家人身安全和财产安全，让民营企业安心放心。深入开展清理拖欠民营企业、中小企业账款专项行动，继续清理规范工程建设领域保证金。发挥各类金融机构优势，完善民营企业信用评级制度，鼓励银行向民营企业发放中长期贷款，推动债券品种创新，扩大优质企业债发行规模，实施好民营企业债券融资支持工具，研究并推动设立民营企业股权融资支持工具，多渠道破解融资难融资贵问题。推进守信激励创新，探索拓展“信易+”服务项目，实现信用惠民便企。对排斥限制民营企业招投标问题开展专项整治。
　　五是深化农业供给侧结构性改革。毫不放松抓好粮食生产，全面落实粮食安全省长责任制，深入推进优质粮食工程，确保粮食播种面积稳定在16.5亿亩。全面完成粮食生产功能区和重要农产品生产保护区划定，加强农田水利建设，新增高标准农田8000万亩以上。优化品种结构，大力发展紧缺和绿色优质农产品生产，推进农业由增产导向转向提质导向。稳步推进耕地轮作休耕制度试点。实施质量兴农战略规划，建设质量兴农政策、评价、考核和工作体系。持续推进农业绿色发展，推进建设农业绿色发展先行区。加快农业科技改革创新，大力发展现代种业，加强先进适用技术推广，实施地理标志农产品保护工程，推进农业全程机械化。加快构建乡村产业体系，深入推进农村一二三产业融合发展，壮大县域经济。创新发展乡村特色产业和新型服务业，支持特色农产品优势区、现代农业产业园、农业产业强镇建设，大力培育发展农业产业化国家重点龙头企业，积极培育家庭农场、农民合作社等新型农业经营主体，加快培育发展农村新产业新业态。完善稻谷和小麦最低收购价政策，完善玉米和大豆生产者补贴政策，进一步提高大豆补贴标准，多途径扩大大豆种植面积。扩大政策性农业保险改革试点，创新和加强农村金融服务。毫不松懈做好非洲猪瘟防控工作，稳定畜禽产品生产供应。健全化肥、农药等重要农资储备制度。加快建立符合国际规则的新型农业支持保护政策体系。
　　（二）促进形成强大国内市场。坚持办好自己的事，着眼更好满足人民日益增长的美好生活需要，大力培育发展国内市场，实现经济良性循环和供需动态平衡。
　　一是加快传统产业改造提升。制定加快传统产业改造提升促进形成强大国内市场的实施意见，修订[产业结构调整指导目录](https://www.pkulaw.com/chl/9079abf49b35172cbdfb.html)。强化创新、土地、人才等要素保障，为制造业企业技术改造和转型升级提供中长期资金供给。加强重大技术装备补短板，推动首台（套）装备示范应用。加强质量和品牌建设，强化标准引领，着力提升产品品质。改造提升重大产业基地，培育发展现代产业集群。组织实施新一轮技术改造升级工程，运用互联网、大数据、人工智能等新技术和先进适用绿色工艺、技术、装备改造传统产业，促进传统产业安全、绿色、集聚、高效发展。
　　二是大力培育新兴产业。优化新业态新模式的监管体制，深化新兴产业政银企合作，在新一代信息技术、高端装备、生物技术、新材料等重点领域，优先培育和大力发展一批战略性新兴产业集群。加快建立数字经济政策体系，制定实施新时期“互联网+”行动，实施数字经济、“互联网+”重大工程，建设人工智能创新应用先导区，持续推进大数据综合试验区建设。加快5G商用步伐和IPv6规模部署，加强人工智能、工业互联网、物联网等新型基础设施建设和融合应用。研究制定新时期“宽带中国”战略，加强新一代信息基础设施建设。研究制定生物经济发展战略纲要和促进生物产业发展的若干政策，深入实施生物产业倍增工程，促进生物技术和信息技术融合发展。加大通用航空产业发展步伐。
　　三是聚焦关键领域促进有效投资。制定基础设施高质量发展实施方案，推进落实保持基础设施领域补短板力度有关举措。安排中央预算内投资5776亿元，比上年增加400亿元。调整优化中央预算内投资结构，重点用于“三农”建设、重大基础设施建设、创新驱动和结构调整、保障性安居工程、社会事业和社会治理、节能环保与生态建设等方面。加大对在建项目的资金保障，鼓励地方政府盘活存量财政资金，重点用于在建基础设施项目，防止出现“半拉子”工程。建立健全吸引民间资本投资重点领域长效机制，引导民营企业参与国家重大战略和补短板项目建设。鼓励民间资本采取混合所有制、投标联合体等多种方式参与PPP项目。加快推动川藏铁路、沿江高铁、北煤南运通道体系、跨区输电通道、西南水电等重大项目的规划建设，支持京津冀、长江三角洲、粤港澳大湾区、山东半岛、北部湾等重点城市群加快推进城际铁路、市域（郊）铁路规划建设，抓好三峡库区后续工程建设，抓紧推进三峡枢纽水运新通道和葛洲坝航运扩能工程等重大项目前期工作，规划建设西部陆海新通道，加快建设天然气管网设施互联互通“全国一张网”。推进枢纽机场新建、迁建和改扩建工程，有序建设支线机场和通用机场，加快推进北京大兴国际机场建设和运营筹备工作，确保9月底前建成投运。大力推进综合客运枢纽、物流枢纽建设，制定国家物流枢纽网络建设实施方案，支持集疏港铁路等建设，打通运输“最后一公里”。
　　四是积极培育消费惠民新增长点。研究制定居民增收三年行动方案。落实好个人所得税基本减除费用标准和6个专项附加扣除政策，深入推进城乡居民增收试点工作，推动实施技能人才、新型职业农民、科研人员等重点群体增收激励计划，完善事业单位岗位绩效工资制度，进一步增强居民消费能力。制定出台促进汽车、家电等热点产品消费的措施，畅通城乡双向联动销售渠道，开展品牌消费、品质消费“双品网购节”系列活动，挖掘农村网购和乡村旅游消费潜力。实施质量强国战略，推动产品和服务质量提升。开展打击假冒伪劣三年行动，净化市场消费环境。深入实施“同线同标同质”工程，加强重要商品质量追溯体系建设，改造提升重点城市步行街，吸引高端商品和服务等境外消费回流。推动各类应用电子产品智能化升级，进一步扩大和升级信息消费。创新激励政策，强化市场监管，扩大绿色消费。加快教育、育幼、养老、家政、医疗、文化、旅游等服务业发展。丰富文化和旅游产品及服务供给，创新业态和消费模式。研究制定健康产业发展行动纲要，加大体育消费设施建设力度。落实带薪年休假制度。出台实施大力发展老年人照护服务的政策措施，开展家政服务标准化试点示范建设，加大家政人员培训力度。推进海南国际旅游消费中心、平潭国际旅游岛和横琴国际休闲旅游岛建设，组织实施“三区三州”等深度贫困地区旅游基础设施改造升级行动计划（2018－2020年）。加强重点旅游景区快速交通、步行道、停车场等基础设施和公共服务设施建设，规范主题公园健康发展，支持邮轮、旅居车等消费发展。研究制定促进东北地区寒地冰雪经济发展的指导意见。实施社会办医疗机构跨部门联合审批。
　　

　　图表：
　　关于2018年国民经济和社会发展计划执行情况与2019年国民经济和社会发展计划草案的报告 新华社发
　　五是切实稳定市场预期。积极主动加强重大政策解读，创新宣传手段，及时向各类市场主体精准解读政策精神。及时妥善化解可能影响预期的重大风险隐患，密切关注舆情走势，及时回应社会关切，强化重特大突发事件应急处置。做好第四次全国经济普查数据采集、发布和解读工作。密切跟踪分析重要商品市场供需和价格变化，做好市场保供和物价稳定工作，加强产供储销运调节，严厉打击囤积居奇、哄抬物价等不法行为。
　　（三）坚决打好三大攻坚战。针对突出问题，集中力量、分类推进、精准施策，打好重点战役，全力攻坚、务求实效。
　　一是打好防范化解重大风险攻坚战。坚决贯彻总体国家安全观，健全维护国家经济安全的体制机制，强化重大风险监测研判预警，守住不发生系统性风险的底线。坚持结构性去杠杆基本思路，把握好节奏和力度，推动市场化债转股签约项目尽快落地，加强国有企业资产负债约束，建立企业债务风险监测、预警体系。加强对股市、债市、汇市资金流动和风险的监管与处置，防范金融市场异常波动和共振。完善资本市场基本制度，加强互联网金融等领域监管，有序化解影子银行风险。弥补监管制度短板，尽快出台金融控股公司监管办法。稳妥处理地方政府债务风险，坚决遏制地方政府隐性债务增量、有序推进存量化解，支持融资平台公司市场化转型。坚持房子是用来住的、不是用来炒的定位，稳妥实施房地产市场平稳健康发展长效机制方案，落实城市主体责任制，稳地价、稳房价、稳预期，坚决防范化解房地产市场风险。坚持租购并举，完善住房市场体系和住房保障体系，努力解决城镇中低收入居民和新市民住房问题。改革住房公积金制度。在人口净流入的大中城市加快发展住房租赁市场特别是长期租赁市场。
　　二是打好精准脱贫攻坚战。扎实推进打赢脱贫攻坚战三年行动，集中力量支持“三区三州”等深度贫困地区和特殊贫困群体脱贫攻坚，重点解决好实现“两不愁三保障”面临的突出问题。深入实施产业、就业、生态、教育、健康、社会保障等扶贫，实施残疾人脱贫行动。进一步落实脱贫攻坚责任制，完善脱贫攻坚考核监督评估机制。研究建立促进困难群众稳定脱贫和防范返贫的长效机制，减少和防止贫困人口返贫。对摘帽后的贫困县通过实施乡村振兴战略，巩固脱贫成果、提升发展水平。研究解决收入水平略高于建档立卡贫困户的群体缺乏政策支持等新问题。全年再减少农村贫困人口1000万人以上，基本完成“十三五”易地扶贫搬迁规划建设任务。
　　三是打好污染防治攻坚战。坚守阵地、巩固成果，不放宽放松，更不走回头路，协同推进经济高质量发展和生态环境高水平保护。聚焦打赢蓝天保卫战、柴油货车污染治理、城市黑臭水体治理、长江保护修复、渤海综合治理、农业农村污染治理、水源地保护等七大战役，加大工作和投入力度。加强污染防治重大科技攻关。改革创新环境治理方式，制定构建政府为主导、企业为主体、社会组织和公众共同参与的环境治理体系的意见，落实企业污染防治主体责任和环保责任。深入推进生态环境保护督察，帮助企业制定环境治理解决方案。推进钢铁行业超低排放改造，持续稳步推进煤电超低排放和节能改造，促进煤电高效、清洁、高质量发展。优化调整货物运输结构，提升煤炭、矿石、焦炭等大宗货物铁路、水路运输比例。实施城市污水处理提质增效三年行动，全面推进城市生活垃圾分类工作，研究制定进一步加强塑料污染防治的意见。组织实施“无废城市”建设试点和废铅蓄电池污染防治行动。大力推进能源节约和高效利用，促进重点地区煤炭消费减量替代和清洁高效利用。有效推进北方地区冬季清洁取暖，集中资源推进京津冀及周边地区、汾渭平原散煤治理。加强天然气产供储销体系建设，按照“以气定改”原则有序实施煤改气等措施。支持清洁能源多元化应用，提高可再生能源占能源消费比重，建立可再生能源电力消纳利用长效机制。加强生态系统保护和修复，加强荒漠化、石漠化等治理。加大生物多样性保护力度。继续开展退耕还林还草还湿。全面开展生态保护红线勘界定标，建设生态保护红线监管平台。完善多元化的横向生态补偿机制。推进大规模国土绿化，提升森林、草原、湿地等生态系统质量，深化国家公园体制改革。深入实施河长制湖长制，持续推进重点流域综合治理。严格管控围填海。建立健全绿色生产、消费的法律制度和政策体系，研究制定建立健全绿色低碳循环发展经济体系的指导意见，推进资源循环利用基地建设。实施国家节水行动，提高水资源管理水平和利用效率。推广绿色建筑、绿色快递包装。加快构建市场导向的绿色技术创新体系，发布绿色产业指导目录（2019年版），推动绿色高效制冷。改革完善环境经济政策，健全排污权交易制度，大力发展绿色金融。积极推广合同能源管理、合同节水管理、环境污染第三方治理模式，加快实施绿色制造工程，促进节能环保等绿色产业发展。开展绿色生活创建行动。实施应对气候变化国家战略，加快完善全国碳市场制度体系，积极参与全球气候治理。
　　（四）加快建设创新型国家。落实创新驱动发展战略，提高大众创业、万众创新水平，促进军民协同创新，全面提升创新能力和效率。
　　一是加强国家创新体系建设。研究谋划新一轮全面创新改革试验。持续推进科技体制改革，抓好赋予科研机构和人员更大自主权有关政策的贯彻落实。改革科技创新成果的科学、技术、经济等价值评价制度，推动科研成果加快转化。建立健全以创新、质量、贡献为导向的科技人才评价激励体系。编制新一轮国家中长期科技发展规划。加大对基础研究的稳定持续投入。加快实施国家科技重大专项和科技创新2030-重大项目。构建开放、协同、高效的共性技术研发平台，加快健全需求为导向、企业为主体的产学研一体化创新机制。大力支持民营企业开展原始创新，支持龙头企业包括民营高技术企业牵头重大科技项目，强化企业、科研院所和高校的创新资源整合共享。加强工业强基工程协同攻关，推进产业链上下游一条龙示范应用。抓紧布局国家实验室，重组国家重点实验室体系。完善重大科技项目组织管理。加强国家产业创新中心建设，支持新一批国家自主创新示范区建设，在关键领域新布局建设若干国家制造业创新中心。加快建立京津冀协同创新共同体，支持北京、上海科技创新中心建设，支持北京怀柔、上海张江、安徽合肥3个综合性国家科学中心建设，以科技创新支撑长江经济带发展、长江三角洲区域一体化发展战略，研究和大力支持建设粤港澳大湾区国际科技创新中心并布局建设综合性国家科学中心。
　　二是持续推进创新创业高质量发展。加快落实“双创”升级版重点任务。进一步优化创新创业环境，推动落实各项创业扶持政策，健全公共服务体系，积极发展创业孵化基地等各类载体，打造展示交流和资源共享平台。全面加强知识产权保护，建立覆盖知识产权交易、评估等方面的服务体系，健全知识产权侵权惩罚性赔偿制度。进一步完善支持创业投资发展的相关政策，鼓励发行双创专项金融债券、双创孵化专项债券等。推动国家新兴产业创业投资引导基金高效运营，构建新兴领域创业投资联盟。办好2019年全国“双创”活动周。
　　三是扎实推进军民融合深度发展。深入推进军民融合重大示范项目和军民融合创新示范区建设，推动军民融合产业发展，充分发挥军民融合对培育壮大经济发展新动能的重要支撑作用。采取强有力的措施深入开展经济建设项目贯彻国防要求试点，实现共建共享共用。健全军民融合体制机制，破除“民参军”壁垒，加大“军转民”力度。健全军地两用人才培养机制，畅通军地人才双向流动渠道。深入推进国防军工生产能力建设，进一步优化国民经济动员布局。推动军民融合综合性立法。持续深化军工企业混合所有制改革试点。强化军民科技成果双向转化应用。融合拓展国家军民融合公共服务平台功能。
　　（五）持续深化重点领域改革。全面贯彻落实习近平总书记在庆祝改革开放40周年大会上的重要讲话精神，持续强化改革牵引作用，以增强微观主体活力为重点，推动经济体制改革走深走实。
　　一是深化“放管服”改革。加快完善中国营商环境评价体系，健全评价分析系统，在直辖市、计划单列市、部分省会城市和地级市开展营商环境试评价，推动全国范围营商环境实现较大幅度改善，降低制度性交易成本。持续推动[市场准入负面清单](https://www.pkulaw.com/chl/d8135b05437494cebdfb.html)制度全面实施，建立健全清单动态调整机制，不断完善清单信息公开机制，推动“非禁即入”普遍落实。再推动取消一批行政许可等事项，加快清理各类变相审批和许可，压减规范行政处罚事项，大幅压缩工程建设项目审批时间，压缩开办企业、注册商标、登记财产、获得电力、跨境贸易等事项的办理环节、时限和成本，进一步清理规范各类行政审批中介服务事项，全面推开行业协会商会与行政机关脱钩改革，打造法治化、国际化、便利化的营商环境。深化商事制度改革，积极推进“证照分离”改革全覆盖，深入推行企业注销便利化改革，继续压缩专利审查和商标注册时间。加快健全以“双随机、一公开”为基本手段、以重点监管为补充、以信用监管为基础的新型监管机制，在市场监管领域推进跨部门“双随机、一公开”监管，加强反垄断和反不正当竞争执法。大力推动“互联网+政务服务”，加快“数字政府”建设，抓紧建成全国一体化在线政务服务平台，加快实现一网通办、异地可办。出台国家政务信息系统管理办法，建立完善国家政务信息系统清单管理机制。
　　二是推进国资国企改革。扎实推进国企改革“1+N”政策文件落实落地和各项改革试点经验成果的推广应用，出台改革国有资本授权经营体制方案，深化综合性改革，建立职业经理人等制度，培育世界一流企业。推进重要领域混合所有制改革，完善员工持股等配套政策，加快推进一般竞争性领域国有企业混合所有制改革，积极推动民营企业参与国有企业改革。坚持做强做优做大国有资本，推动国有经济战略性重组和布局优化，加快实现从管企业向管资本转变，改组成立一批国有资本投资公司，组建一批国有资本运营公司，推动国有资本投资、运营公司试点取得更大成效。研究扩大国有资本经营预算实施范围，提高国有资本经营预算调入一般公共预算的比例。继续分批推动划转部分国有资本充实社保基金。深入推进国有企业工资决定机制改革。
　　三是深化重点行业改革。深化石油天然气体制改革，组建国家石油天然气管网公司，实现管输和销售分开。放开油气勘查开采准入限制，积极吸引社会资本加大油气勘查开采力度。深化电力体制改革，推动交易机构独立规范运行，加快推进电力市场化交易，稳步推进电力现货市场建设试点，扩大增量配电业务改革试点。在加强成本监审调查基础上，深入推进第二个监管周期输配电价改革，继续推进石油、天然气、交通运输等重点领域价格改革。深化邮政和烟草体制改革。
　　四是优化要素市场化配置。制定关于完善要素市场化配置的意见，健全要素市场准入、监管、退出制度。深入推进人力资源市场建设，促进人力资源自由有序流动。完善投融资体制机制，加快投融资服务引导转型，持续规范简化投资审批，深化企业投资项目承诺制改革。扩大国有土地有偿使用范围，深化工业用地市场化配置改革试点，推进工业用地一级市场多种方式出让和二级市场有序流转，进一步深入推进农村集体经营性建设用地入市。推进公共资源交易平台整合共享。深化技术要素市场化配置，探索职务科技成果产权制度改革。推进数据信息要素市场化配置，完善个人信息授权和大数据交易制度。
　　五是深化财税金融体制改革。制定应急救援、自然资源等领域中央与地方财政事权和支出责任划分改革方案。健全地方税体系，推进中央和地方收入划分改革，全面实施预算绩效管理。深化增值税改革，逐步建立期末留抵退税制度。在上海证券交易所设立科创板并试点注册制，健全上市公司股份回购制度，进一步完善[上市公司现金分红](https://www.pkulaw.com/chl/7cc7cb356d5d22c1bdfb.html)和退出机制。健全货币政策和宏观审慎政策双支柱调控框架，将更多金融活动、金融市场、金融机构和金融基础设施纳入宏观审慎政策框架。完善全口径外债和跨境资本监管体系。改革完善地方金融监管体制，坚决打击非法金融机构和非法金融活动。
　　（六）推进更高水平对外开放。扎实推进共建“一带一路”，在更深层次、更宽领域、以更大力度推进全方位高水平开放。
　　一是深入推进“一带一路”国际合作。办好第二届“一带一路”国际合作高峰论坛。加强我国与沿线国家的发展规划对接。以基础设施等重大项目建设和产能合作为重点，解决好金融支撑、投资环境、风险管控、安全保障等关键问题，加强文化交流与人才合作，确保更多合作成果落地。深入推进数字丝绸之路建设合作，推动中国－东盟智慧城市网络合作。高质量建设境外经贸合作区、产能合作园区等平台。推动中欧班列高质量发展，加快“丝路电商”全球布局。务实推进与法国、德国、日本、新加坡等国家第三方市场合作和创新合作，积极推进与太平洋岛国的经贸、旅游等务实合作。加快沿边开发开放步伐，推动沿边重点开发开放试验区和跨境经济合作区建设。支持东北地区打造我国向北开放的重要窗口和东北亚地区合作的中心枢纽。
　　二是着力稳外贸。进一步压缩整体通关时间，降低进出口合规成本，大力推进“经认证的经营者”（AEO）国际互认合作，提升贸易便利化水平。加大对出口信用保险保单融资、出口退税账户质押融资等政策支持力度，推动出口市场多元化。办好第二届中国国际进口博览会，增加先进技术设备、关键零部件以及短缺的能源资源、农产品进口，促进对外贸易平衡发展。优化进出口结构，巩固传统竞争优势，加快培育以技术、标准、品牌、质量、服务为核心的综合竞争优势，促进服务贸易创新发展，加快服务外包转型升级，积极发展跨境电商，推动外贸向质量效益型转变。
　　三是加大吸引外资力度。出台实施[外商投资法](https://www.pkulaw.com/chl/6a88714068b3724dbdfb.html)，缩减外商投资准入负面清单，全面清理取消负面清单以外领域针对外资设置的准入限制。稳步推进金融业开放，吸引更多长期资金流入，有效发挥国际长期资本的作用。制定鼓励外商投资产业目录，扩大鼓励外商投资范围，吸引跨国公司来华投资并设立生产研发基地。健全外资项目服务机制，推动一批规模大、示范性强的重点项目落地。完善外资安全审查机制。稳步推进外债登记制管理改革，提升监管水平。推进国家级经开区、高新区等各类开发区创新提升，增强辐射带动作用。研究出台推动自贸试验区进一步扩大开放和创新发展的改革措施。增设上海自贸试验区新片区，将支持自贸试验区及全面创新改革试验区的出入境政策措施推广至全国。深入落实支持海南全面深化改革开放的各项政策举措，高质量建设全岛自贸试验区，探索建设中国特色自由贸易港。
　　四是促进对外投资平稳健康发展。加快制定境外投资条例，完善境外投资服务和监管。推动企业加强合规管理，规范企业境外经营秩序，实行守信联合激励和失信联合惩戒。加强“走出去”公共服务平台建设，建立部门间信息统一归集和共享机制。推进安全风险防范体系建设，全面提高境外安全保障和应对风险能力。
　　五是积极参与国际经贸规则变革完善。积极发展全球伙伴关系，充分利用国际平台加强政策协调，扩大同各国的利益交汇点。积极参与世界贸易组织改革，坚定维护多边贸易体制，主动参与多边贸易规则制定，推动构建公正、合理、透明的国际经贸规则体系，维护支持贸易投资自由化便利化的合作方向。落实阿根廷中美元首会晤共识，稳妥推动中美经贸磋商。推动早日达成区域全面经济伙伴关系协定，加快中欧投资协定谈判和中日韩自由贸易区谈判进程。
　　（七）促进城乡区域协调发展。围绕解决发展不平衡不充分问题，推动乡村振兴和区域发展重大战略落地见效，着力提升新型城镇化质量，缩小城乡区域发展差距。
　　一是持续推进乡村振兴战略。落实农业农村优先发展总方针，积极推动乡村振兴战略规划（2018－2022年）各项任务落地。加快编制村庄规划，推进农村基础设施和公共服务提挡升级，健全多元投入机制，促进路水电气信等城乡基础设施一体规划、互联互通，推动公共服务向农村延伸、社会事业向农村覆盖。大力支持农村饮水安全巩固提升等工程建设。抓好农村人居环境整治三年行动，重点推进“厕所革命”，做好农村生活垃圾污水治理，推动垃圾分类和资源化利用，建设美丽乡村。加快推进农村水土流失综合治理，实施生态清洁小流域建设。完善县乡村物流基础设施网络，加强农产品物流骨干网络和冷链物流体系建设，促进重要市政设施城乡共用。实施村庄基础设施建设工程，新建改建农村公路20万公里。全面深化农村改革，深化农村土地征收制度改革，稳慎推进农村宅基地制度改革，推进房地一体的农村集体建设用地和宅基地使用权确权登记颁证，加快盘活利用闲置宅基地和闲置农房，推进农村集体产权制度改革。深化农垦改革发展。
　　二是扎实推进以人为核心的新型城镇化。建立健全城乡融合发展体制机制和政策体系，推进城乡融合发展。推动落实1亿非户籍人口在城市落户目标，加快推进户籍制度改革，推进租赁房屋的常住人口在城市公共户口落户。加快城市群一体化体制机制建设，增强中心城市辐射带动力，加强交通设施联通，优化产业布局。持续提升城市品质，推进城区老工业区、城镇人口密集区危险化学品生产企业等搬迁改造，有序推动城市老旧小区改造，支持加装电梯和无障碍环境建设，积极打造新型智慧城市，推进地下综合管廊、海绵城市和城市排水防涝设施建设。规范有序建设特色小镇。加快建立多元可持续的城镇化资金保障机制。
　　三是着力推动区域协调发展。制定西部开发开放新的政策措施，推进西部大开发形成新格局，推动西部地区生态环境、营商环境、开放环境、创新环境明显改善。支持东北地区深化改革创新推动高质量发展，加快落实优化营商环境、深化国企改革、促进民营经济发展等政策举措。支持中部地区发挥优势，夯实制造业基础，有力有序承接国内外产业转移。支持中西部和东北地区建设科技成果转移转化示范区。推进实施淮河生态经济带发展规划。加大力度支持革命老区、民族地区、边疆地区、贫困地区加快发展，深入实施兴边富民行动，积极促进资源型地区经济转型发展。推动国家级新区高质量发展，积极推动和支持临空经济示范区、自主创新示范区等平台建设。制定坚持陆海统筹、加快建设海洋强国政策及规划，推动海洋经济高质量发展。
　　四是深入实施国家重大区域战略。促进京津冀协同发展，积极稳妥有序推进北京非首都功能疏解，出台实施雄安新区相关规划及改革开放配套实施方案，推进交通、生态、公共服务等基础设施项目建设，持续推动北京城市副中心建设，建好首都水源涵养功能区与生态环境支撑区。深入推动长江经济带发展，按照统筹山水林田湖草系统治理的思路，扎实推进“三水共治”和生态环境治理“4+1”工程，发挥铁路、公路、水运各自优势整体设计综合运输体系。将长江三角洲区域一体化发展上升为国家战略，制定实施发展规划纲要。编制粤港澳大湾区产业发展、基础设施、生态环境等专项规划，支持深圳前海、广州南沙、珠海横琴等粤港澳合作发展平台建设。
　　（八）切实保障和改善民生。坚持以人民为中心的发展思想，突出保基本兜底线，精心做好各项民生工作，实施好更具有普惠性和可持续性的社会政策。
　　一是扎实做好就业优先工作。加强对美贸易重点企业用工监测，完善风险应对预案，落实好受影响企业的援企稳岗等措施。扎实做好高校毕业生、退役军人、农民工等重点群体就业工作，加强对城镇各类就业困难人员的就业帮扶。深入实施高校毕业生就业创业促进计划和基层成长计划，启动实施三年百万青年见习计划。加强对灵活就业、新就业形态的支持，鼓励各地加快建设创业孵化载体，深入推进返乡创业试点示范。不断健全职业技能培训制度，加快推行终身职业技能培训制度。健全技术工人职业发展机制。组织实施公共职业技能培训基础平台建设行动方案。加快产教融合实训基地建设。大力弘扬工匠精神，积极推行企业新型学徒制。推进农民工职业技能提升计划等专项培训。继续实施阶段性降低失业保险费率政策，实质性扩大失业保险基金稳就业支出，全面落实失业保险基金稳岗返还政策。实施职业技能提升行动，从失业保险基金结余中拿出1000亿元，用于1500万人次以上的职工技能提升和转岗转业培训。支持困难企业开展职工在岗培训，加强失业人员职业技能培训或创业培训。对招用农村贫困人口、城镇登记失业半年以上人员的各类企业，三年内给予定额税费减免。加大对残疾人等就业困难人群帮扶力度，确保零就业家庭动态清零。
　　二是进一步完善社会保障制度和政策。统筹推进多层次社会保障体系建设，推进以国家政务服务平台为统一入口的社会保险公共服务平台建设。研究制定建立退休人员基本养老金合理调整机制。推进贫困人口基本养老保险制度全覆盖。落实企业职工基本养老保险基金中央调剂制度，加快完善养老保险省级统筹，落实降低社会保险费率相关工作。全面推进建立统一的城乡居民基本医疗保险和大病保险制度。扩大长期护理保险制度试点。加强医疗保障基金监管。进一步推进跨省异地就医直接结算。完善农村低保制度，健全低保对象认定办法。完善失业保险制度，研究解决新业态从业人员中无工作单位人员的职业伤害保障问题。落实特困人员救助供养制度，加大临时救助力度，优化救助方式和审核审批程序，认真执行社会救助和保障标准与物价上涨挂钩联动机制。持续开展农村危房改造，继续做好棚户区改造工作，进一步规范发展公租房。
　　三是深入推进公共服务补短板强弱项提质量。制定国家基本公共服务标准，推动各地区各领域标准规范做好衔接，实施社会领域公共服务补短板、强弱项、提质量行动方案。出台实施促进3岁以下婴幼儿照护服务发展的指导意见，着力增加托育服务有效供给。坚持教育优先发展。积极支持优质普惠学前教育资源扩容建设，坚持公办民办并举，在持续办好公办幼儿园的同时，引导社会力量多举办普惠性幼儿园，加快完善普惠性民办幼儿园认定标准、补助标准及扶持政策。继续推进城乡义务教育一体化发展，深入实施高中阶段教育普及攻坚计划，积极消除城镇中小学“大班额”。持续改善贫困地区学校基本办学条件，加大对农村贫困地区儿童早期发展的支持力度，强化农村和贫困地区控辍保学工作，加强乡村教师队伍建设。依法支持民办教育发展。科学稳妥推进民族地区双语教育。发展现代职业教育，改革高职院校考试招生办法、办学体制和育人机制，支持企业和社会力量兴办职业教育，加快学历证书和职业技能等级证书互通衔接。加快“双一流”建设，促进高等教育内涵式发展。实施好教育现代化推进工程。2019年，九年义务教育巩固率预期达到94.6%，高中阶段教育毛入学率预期达到89.4%，高职院校扩招100万人，普通高等教育本专科招生预期达到870万人，研究生招生预期达到93.3万人。深化医药卫生体制改革。积极推动建立分级诊疗制度、现代医院管理制度、全民医疗保障、药品供应保障、医疗卫生行业综合监管五项制度。发展“互联网+医疗健康”，加快建立远程医疗服务体系。稳步推进区域医疗中心规划建设，加强乡村医生队伍建设。加强重大疾病防治，继续实施疑难病症诊治能力提升和中医药传承创新工程。加快儿童药物研发。加强罕见病用药保障。深化医保支付方式改革，优化医保支出结构。坚持预防为主，加强公共卫生服务、职业病和地方病防治能力建设。抓好青少年近视防治。完善生育配套政策。加快完善疫苗药品监管长效机制。进一步加强医德医风建设和医疗卫生领域失信治理。开展社会办医示范行动，制定促进健康产业高质量发展行动纲要。加强居民身心健康教育和自我健康管理。加强城乡养老设施建设，积极引导民间资本和外资进入养老服务业，大力发展养老特别是社区养老服务业。推进实施社会服务兜底工程，提升老年人、残疾人、困境儿童、农村留守儿童、精神障碍患者等困难群体服务设施建设水平。切实加强妇女、未成年人、残疾人等社会群体权益保护。启动实施积极应对人口老龄化中长期规划。推动大运河文化带建设，积极推进平安故宫、国家美术馆、中国工艺美术馆、国家图书馆国家文献战略储备库等重大文化项目建设。深入推进中华优秀传统文化传承发展，加强文物保护利用改革和历史文化遗迹保护修复。繁荣发展哲学社会科学，建好中国特色新型智库。倡导全民阅读。大力推进实施全国红色旅游经典景区三期总体建设方案。推动冬奥会场馆建设，加大体育设施建设力度。办好2019年北京世界园艺博览会。严格落实安全生产责任制，完善安全监管体制，抓好食品药品、交通等安全。
　　进一步支持香港、澳门融入国家发展大局，加快推进粤港澳大湾区规划建设，实施好港澳居民证件便利化、进一步便利港澳居民到内地创业就业、降低粤港澳通信漫游费用等政策措施。支持香港、澳门参与国家经济高质量发展和新一轮高水平开放，努力把香港、澳门打造成国家双向开放的重要区域。支持香港提升国际金融、航运、贸易中心地位，促进香港同内地加强科技合作，支持香港建设国际创新科技中心。支持澳门加强世界旅游休闲中心、中葡商贸合作服务平台建设，打造中医药科技产业发展平台，支持澳门打造以中华文化为主流、多元文化共存的交流合作基地。扩大海峡两岸交流合作、深化两岸融合发展，持续落实促进两岸经济文化交流合作的31条措施，推出更多惠及台湾同胞的政策措施，为台胞台企在大陆实现更好发展创造条件。
　　各位代表，做好2019年经济社会发展工作意义重大，任务艰巨。我们要更加紧密地团结在以习近平同志为核心的党中央周围，高举中国特色社会主义伟大旗帜，以习近平新时代中国特色社会主义思想为指导，按照党中央、国务院决策部署，自觉接受全国人大的监督，虚心听取全国政协的意见和建议，敢于担当、真抓实干，自力更生、艰苦奋斗，改革创新、攻坚克难，促进经济社会平稳健康可持续发展，以优异成绩庆祝中华人民共和国成立70周年！

第十三届全国人民代表大会财政经济委员会关于2018年国民经济和社会发展计划执行情况与2019年国民经济和社会发展计划草案的审查结果报告
　　（2019年3月12日第十三届全国人民代表大会第二次会议主席团第二次会议通过）

　　十三届全国人大二次会议主席团：
　　第十三届全国人民代表大会第二次会议审查了国务院提出的《关于2018年国民经济和社会发展计划执行情况与2019年国民经济和社会发展计划草案的报告》和2019年国民经济和社会发展计划草案。全国人民代表大会财政经济委员会在对计划报告和计划草案初步审查的基础上，根据各代表团和有关专门委员会的审查意见，又作了进一步审查。国务院根据审查意见对计划报告作了修改。现将审查结果报告如下。

　　**一、**2018年计划执行情况总体良好
　　财政经济委员会认为，2018年国民经济和社会发展计划执行情况总体良好。面对错综复杂的国际环境和艰巨繁重的国内改革发展稳定任务，国务院和地方各级人民政府在以习近平同志为核心的党中央坚强领导下，坚持以习近平新时代中国特色社会主义思想为指导，全面贯彻党的十九大精神，按照党中央决策部署和十三届全国人大一次会议各项决议要求，坚持稳中求进工作总基调，落实高质量发展要求，有效应对外部环境深刻变化，迎难而上、扎实工作，经济保持总体平稳、稳中有进的发展态势。从计划指标完成情况看，对照十三届全国人大一次会议批准的2018年国民经济和社会发展计划，全部65个指标中绝大多数符合或好于预期。各项重点任务完成情况较好，供给侧结构性改革深入推进，三大攻坚战开局良好，改革开放力度加大，稳妥应对中美经贸摩擦，人民生活持续改善，保持了经济持续健康发展和社会大局稳定。成绩来之不易。同时也要看到，经济运行稳中有变、变中有忧。外部环境更趋复杂严峻，风险挑战增多；内需增长放缓并向供给侧传导，经济下行压力有所加大；自主创新能力尚不能满足转型升级和高质量发展需要；实体经济成本偏高，负担较重，企业融资难融资贵问题仍然突出；长期积累的金融、地方政府债务、房地产等风险依然存在；营商环境有待进一步优化，市场预期和投资者信心有待提振；区域分化仍在持续；就业结构性矛盾突出，城乡居民收入增幅放缓，教育、医疗、养老等社会领域改革发展和公共服务短板依然明显。对此，要增强忧患意识，加强政策统筹和工作协调配合，有针对性地加以解决。

　　**二、**2019年计划报告和计划草案总体可行
　　财政经济委员会认为，国务院提出的2019年计划报告和计划草案，符合中央经济工作会议精神，符合“十三五”规划纲要要求，符合我国经济社会发展实际，指导思想明确，主要目标和工作安排总体可行，建议第十三届全国人民代表大会第二次会议批准国务院提出的《关于2018年国民经济和社会发展计划执行情况与2019年国民经济和社会发展计划草案的报告》，批准2019年国民经济和社会发展计划草案。

　　**三、**做好2019年计划执行工作的建议
　　2019年是新中国成立70周年，是全面建成小康社会、实现第一个百年奋斗目标的关键之年，要在以习近平同志为核心的党中央坚强领导下，以习近平新时代中国特色社会主义思想为指导，全面贯彻党的十九大和十九届二中、三中全会精神及中央经济工作会议精神，按照统筹推进“五位一体”总体布局和协调推进“四个全面”战略布局的要求，坚持稳中求进工作总基调，坚持新发展理念，坚持推动高质量发展，坚持以供给侧结构性改革为主线，坚持深化市场化改革、扩大高水平开放，加快建设现代化经济体系，继续打好三大攻坚战，着力激发微观主体活力，创新和完善宏观调控，统筹推进稳增长、促改革、调结构、惠民生、防风险、保稳定工作，保持经济运行在合理区间，进一步稳就业、稳金融、稳外贸、稳外资、稳投资、稳预期，提振市场信心，增强人民群众获得感、幸福感、安全感，保持经济持续健康发展和社会大局稳定，为全面建成小康社会收官打下决定性基础。为此，财政经济委员会提出以下建议：
　　（一）进一步加强和改善宏观调控，提升政策实施效果。增强宏观调控的前瞻性、针对性和灵活性，强化逆周期调节，适时预调微调。积极的财政政策要加力提效，实施好更大规模减税降费，确保政策落实到位，确保主要行业税负明显降低，确保所有行业税负只减不增。稳健的货币政策要松紧适度，保持流动性合理充裕，改善货币政策传导机制，提升金融服务实体经济效能。抓好部门协调配合和政策协同配套，把握好调控的节奏和力度，强化政策针对性和可操作性，确保实现预期效果。促进形成强大的国内市场，持续释放内需潜力。提高城乡居民收入水平，改善消费环境，促进居民消费；发挥投资关键作用，注重拉动有效投资，提高补短板资金使用效率。加强和改进预期管理，提高政策透明度，稳定市场预期，提振社会信心。
　　（二）继续深化供给侧结构性改革，着力振兴实体经济。按照“巩固、增强、提升、畅通”八字方针，更多运用市场化、法治化手段推进供给侧结构性改革进一步深化。高度关注制造业占经济比重持续下降问题，推动传统产业改造提升，促进战略性新兴产业加快发展，坚定不移建设制造强国。有效降低企业成本负担，重点减轻制造业和小微企业负担。加大对乱收费查处和整治力度。明显降低企业社保缴费负担，稳定现行缴费方式。着力缓解企业融资难融资贵，完善商业银行差异化考核和激励机制，发展多层次资本市场，提高直接融资特别是股权融资比重。加快治理政府部门拖欠企业以及企业间相互拖欠账款问题。优化环保、消防、税务、市场监管等执法方式，降低企业合规成本。
　　（三）巩固成果、精准施策，坚决打好三大攻坚战。坚决打好防范化解重大风险攻坚战，着重防范化解金融、地方政府债务、房地产等领域风险，牢牢守住不发生系统性风险的底线。打好精准脱贫攻坚战，重点解决好实现“两不愁三保障”面临的突出问题，加大“三区三州”等深度贫困地区和特殊贫困群体脱贫攻坚力度，因地制宜解决易地扶贫搬迁后持续发展问题，巩固脱贫成果，防止脱贫人口返贫。保持加强生态文明建设的战略定力，打好污染防治攻坚战，加大工作力度，努力完成“十三五”规划纲要节能环保指标，确保主要污染物排放总量持续减少，生态环境质量持续改善。
　　（四）增强创新能力，提高经济发展质量。落实创新驱动发展战略，完善国家创新体系，着力解决资源配置重复、科研力量分散、创新主体功能定位不清晰等突出问题。采取积极有效措施进一步提高研究与试验发展经费投入强度，努力实现“十三五”规划纲要提出的目标要求。加大基础研究和前沿领域科研投入，加快实施国家科技重大专项和科技创新2030－重大项目，加大关键核心技术攻关力度。强化企业创新主体地位，鼓励引导企业加大研发投入。推动科技成果转化和产业化，落实好创新导向的普惠性政策，促进高技术产业发展。
　　（五）协同推进乡村振兴和新型城镇化战略，促进区域协调发展。坚持农业农村优先发展，调整优化农业结构，推动农村各产业融合发展，促进农民增收。加快落实农村承包地“三权”分置改革配套政策，全面推开土地征收制度改革和农村集体经营性建设用地入市改革，推广农村宅基地制度改革试点成果。把乡村振兴战略与新型城镇化紧密结合起来，提高新型城镇化质量，着力解决好农村转移人口市民化问题。推进城乡融合发展，促进人才、资源、要素合理配置和流动，提高城乡基本公共服务均等化水平。深入实施国家重大区域战略，加快构建区域协调发展新机制，增强区域发展协调性和平衡性。
　　（六）继续深化重点领域改革，推进更高水平对外开放。以增强市场主体活力为重点，推进各领域改革不断深化，增强发展内生动力。加快国资国企改革，坚持政企分开、政资分开和公平竞争原则，推动国有经济战略性重组和布局优化，深化电力、油气、铁路等重点行业改革。积极支持民营经济发展，营造法治化营商环境，切实保护民营企业合法权益，激发和保护企业家精神。深化财税金融体制改革。推进财政事权和支出责任划分改革，健全地方税体系。改革优化金融体系结构，完善资本市场基础制度，加快推进上交所设立科创板并试点注册制改革，扩大资本市场对外开放。切实转变政府职能，大力简政放权，加强事中事后监管，强化竞争政策基础性地位。在更高水平推进全方位对外开放，全面实施准入前国民待遇加负面清单管理模式，营造内外资一视同仁、公平竞争的市场环境，扎实推进共建“一带一路”。做好应对中美经贸摩擦工作，积极参与国际经贸规则制定和全球经济治理体系变革，促进贸易和投资自由化便利化。
　　（七）进一步保障和改善民生，推动社会事业全面发展。落实好就业优先政策，着力解决结构性失业问题，重点抓好高校毕业生、农民工、退役军人等群体就业，解决好结构调整中转岗人员再就业问题。继续推进城乡义务教育均衡发展，增加对学前教育、职业教育、民族教育等的投入，着力保障好农村留守儿童和农民工随迁子女教育。完善养老保险基金中央调剂制度，确保养老金足额按时发放。全面建立全国统一的城乡居民基本医保和大病保险制度，进一步推进跨省异地就医直接结算，把更多救命救急的好药纳入医保，着力解决群众“看病难、看病贵”问题。积极应对人口老龄化趋势，加快发展养老事业和产业，努力解决养老难问题。下更大力气抓好安全生产、食品药品安全等问题，落实主体责任，消除各类安全隐患。加强和创新社会治理，保持社会和谐稳定。
　　以上报告，请予审议。

第十三届全国人民代表大会财政经济委员会
　　2019年3月12日

© 北大法宝：（[www.pkulaw.com](https://www.pkulaw.com/)) 专业提供法律信息、法学知识和法律软件领域各类解决方案。北大法宝为您提供丰富的参考资料，正式引用法规条文时请与标准文本核对。
欢迎查看所有[产品和服务](https://www.pkulaw.com/helps/2.html)。法宝快讯：[如何快速找到您需要的检索结果？](https://www.pkulaw.com/helps/70.html)[法宝](https://www.pkulaw.com/helps/69.html)V6有何新特色？

扫描二维码阅读原文

**原文链接：**<https://www.pkulaw.com/chl/a8fc9f9d8abe314bbdfb.html>
